# Distinct characteristics of central serous chorioretinopathy according to gender

**DOI:** 10.1038/s41598-022-14777-8

**Published:** 2022-06-22

**Authors:** Seigo Yoneyama, Ayumi Fukui, Yoichi Sakurada, Nobuhiro Terao, Taiyo Shijo, Natsuki Kusada, Atsushi Sugiyama, Mio Matsubara, Yoshiko Fukuda, Wataru Kikushima, Ravi Parikh, Fumihiko Mabuchi, Chie Sotozono, Kenji Kashiwagi

**Affiliations:** 1 Department of Ophthalmology, University of Yamanashi, Shimokato 1110, Chuo, Yamanashi 409-3898 Japan; 2 Department of Ophthalmology, Kyoto Prefectural University of Medicine, Kyoto, Japan; 3 New York University School of Medicine, New York, NY USA; 4 Manhattan Retina and Eye Consultants, New York, NY USA

**Keywords:** Medical research, Pathogenesis

## Abstract

To investigate the differences in clinical and genetic characteristics between males and females with central serous chorioretinopathy (CSC). Consecutive 302 patients (mean age; 56.3 ± 11.7, male/female: 249/53) with CSC were evaluated on the initial presentation. All CSC patients underwent fluorescein angiography and indocyanine green angiography (FA/ICGA), swept-source or spectral-domain optical coherence tomography (OCT), and fundus autofluorescence (FAF) to confirm a diagnosis. All patients were genotyped for rs800292 and rs1329428 variants of *CFH* using TaqMan technology. On the initial presentation, female patients were significantly older (p = 2.1 × 10^–4^, female 61.6 ± 12.4 vs male 55.1 ± 11.3) and had thinner subfoveal choroidal thickness (p = 3.8 × 10^–5^) and higher central retinal thickness (p = 3.0 × 10^–3^) compared to males. A descending tract was more frequently seen in males than in females (p = 8.0 × 10^–4^, 18.1% vs 0%). Other clinical characteristics were comparable between the sexes. The risk allele frequency of both variants including *CFH* rs800292 and *CFH* rs1329428 was comparable between males and females (*CFH* rs800292 A allele male 51.2% vs female 47.2%, *CFH* rs1329428 T allele male 56.2% vs 52.8%). On the initial presentation, age, subfoveal choroidal thickness and central retinal thickness differ between males and females in eyes with CSC. A descending tract may be a strong male finding in CSC.

## Introduction

Central serous chorioretinopathy (CSC), a common retinal-choroidal disease predominantly seen in males, is characterized by serous detachment of neurosensory retina with or without detachment of retinal pigment epithelium (RPE) in the posterior pole^1^. In most cases, subretinal fluid (SRF) spontaneously resolves and the course was usually self-limiting; however, in some cases, persistent or recurrent SRF results in severe vision loss due to RPE atrophy and/or dysfunction of photoreceptor cells^2^. It has been reported that male gender, testosterone, exposure to exogeneous corticosteroids, and pregnancy are associated with CSC^1^. Several candidate genes have been studied and genome-wide association studies were performed for CSC^3–6^; the complement factor H (*CFH)* gene is known to be variants associated with CSC in different ethnicities^3,7,8^.

Although the etiology of CSC has not been fully understood, the choroidal dysfunction is considered of the primary driver in the pathogenesis of CSC. Improved innovation in retinal imaging enabled the visualization of the choroid and its intrachoroidal structure^9^. In eyes with CSC, increased choroidal thickness is generally seen as well as dilated outer choroidal vessel (pachyvessels) on spectral-domain or swept-source optical coherence tomography (SD-OCT or SS-OCT)^10^. The retinal pigment epithelium (RPE) is considered as the other origin of CSC pathogenesis. Maumenee first described leaks at the level of the RPE seen on fluorescein angiography in eyes with CSC implicating RPE dysfunction as a feature of pathogenesis of CSC^11^.

A study using OCT demonstrated that choroidal thickness was increased in males compared to females in the normal healthy eyes^12^, and a recent study reported that male patients with CSC also had a thicker choroid compared to female patients^13^. Similarly, biological gender differences may play a role in possibly the pathogenesis of CSC and it is important to elucidate the predilection of CSC to classically be described as a condition affecting young to middle aged men in their 30–50 s.

In the present study, we investigated clinical and genetic characteristics of CSC and compared them between male and female CSC patients.

## Results

**Figure 1:**
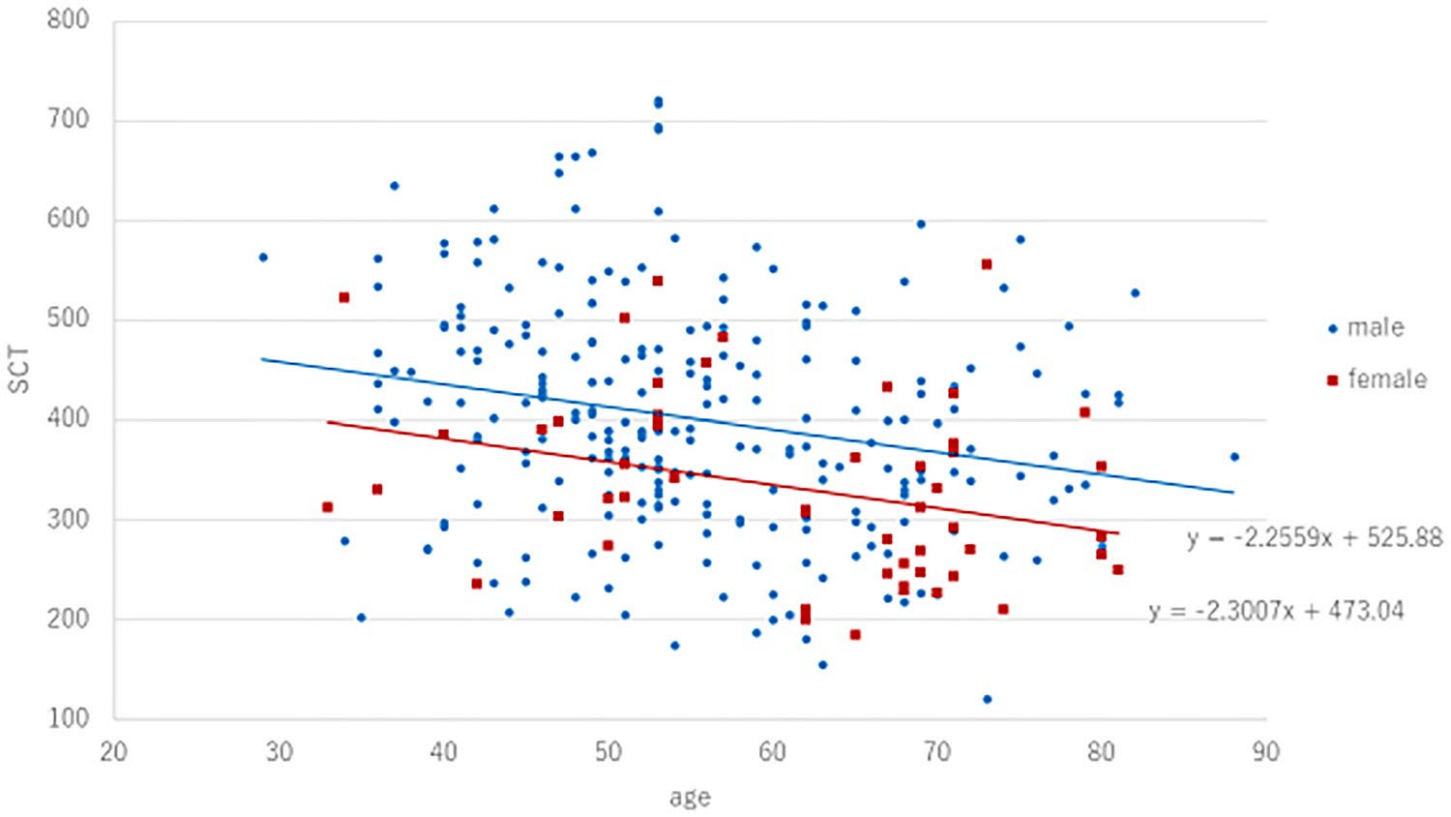
A scatter diagram showing the association between age and subfoveal choroidal thickness. Regardless of gender, subfoveal choroidal thickness decreased by age. The estimated subfoveal choroidal thickness was calculated by 525.88-(Age) × 2.2559 and 473.04-(Age) × 2.3007 in male and female, respectively.

**Figure 2:**
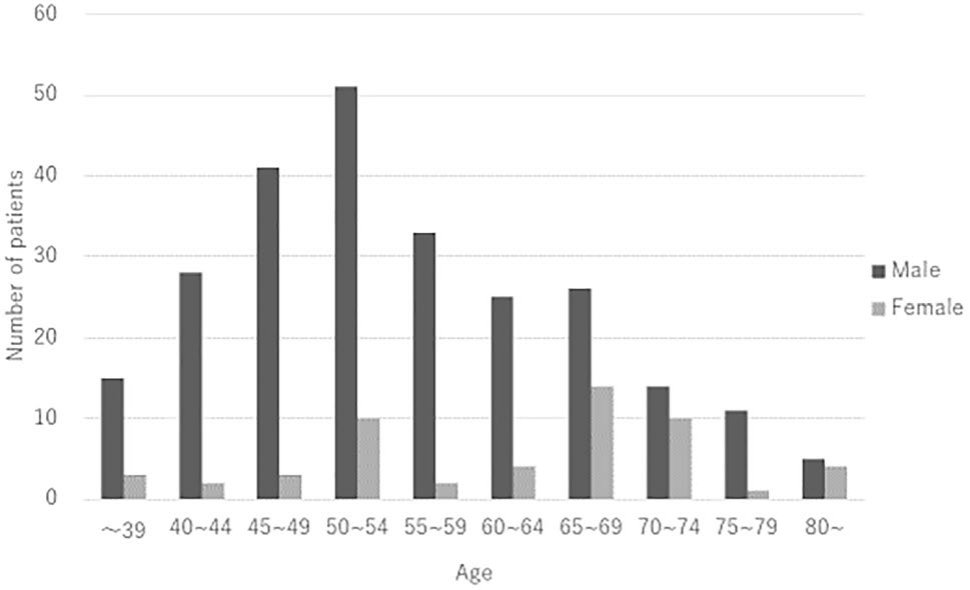
Age distribution by sexes on the initial presentation. In males, the range of 50–54 years (n = 51, 20.5%) was most prevalent followed by the range of 45–49 years (n = 41, 16.5%). In females, the range of 65–69 years (n = 14, 26.3%) was most prevalent followed by the range of 50–54 years(n = 10, 18.9%) and the range of 70–74 years (n = 10, 18.9%).

A total of 302 patients with CSC were included in the present study (male/female: 249/53, mean age: 56.3 ± 11.7). Table 1 shows clinical and genetic characteristics of patients with CSC on the initial presentation. Average subfoveal choroidal and central retinal thickness was 389 ± 114 µm and 327 ± 140 µm, respectively. Table 2 shows the comparison of clinical and genetic characteristics between sexes. Average subfoveal choroidal thickness was 402 ± 115 µm among males compared to 331 ± 92 µm among females (p = 3.8 × 10^–5^). Average central retinal thickness was 314 ± 130 µm among males compared with 384 ± 170 µm among females (p = 3.0 × 10^–3^). Fig. 1 shows a scatter diagram showing the association between age and subfoveal choroidal thickness. In all generations, subfoveal choroidal thickness was estimated to be greater in males than in females. Table 2 shows the comparison of clinical and genetic characteristics between sexes. Compared to males, females had significantly thinner choroids and higher central retinal thickness at baseline. In clinical findings, descending tracts were seen in 18.1% (45/249) males and 0% (0/53) females (p = 8.0 × 10^–4^). Compared with eyes without descending tracts, eyes with descending tracts had significantly thicker choroids (422 ± 133 vs 383 ± 110, p = 0.044, Mann–Whitney U test) and lower central retinal thickness (264 ± 138 vs 337 ± 138, p = 2.8 × 10^–4^ Mann–Whitney U test). The risk allele frequency of *CFH* gene including rs800292 and rs1329428 was not significantly different between sexes. Figure 2 shows the age distribution by sexes. In males, the range of 50–54 years (n = 51, 20.5%) was most prevalent followed by the range of 45–49 years (n = 41, 16.5%). In females, the range of 65–69 years (n = 14, 26.3%) was most prevalent followed by the range of 50–54 years (n = 10,18.9%) and the range of 70–74 years (n = 10, 18.9%).

**Table 1:** Clinical and genetic characteristics of patients with CSC on the initial presentation.

Age	56.3 ± 11.7
Gender (male:female)	249:53
Bilateral involvement	83 (27.5%)
Central retinal thickness (µm)	327 ± 140
Subfoveal choroidal thickness (μm)	389 ± 114
Baseline BCVA (log MAR)	0.21 ± 0.37
Descending tract	45 (14.9%)
Pachydrusen	82 (27.2%)
Fibrin	19 (6.3%)
Pigment epithelial detachment	41 (13.6%)
***CFH* I62V (rs800292)**	
GG	74 (24.5%)
GA	151 (50.0%)
AA	77 (25.5%)
A allele (risk allele) frequency	50.5%
***CFH* rs1329428**	
CC	53 (17.6%)
CT	162 (53.6%)
TT	87 (28.8%)
T allele (risk allele) frequency	55.6%

**Table 2:** Comparison of clinical and genetic characteristics between sexes.

	Male (n = 249)	Female (n = 53)	Univariate p-value
Age	55.1 ± 11.3	61.6 ± 12.4	2.1 × 10^–4^
Bilateral involvement	72 (28.9%)	11 (20.8%)	0.23
Central retinal thickness (µm)	314 ± 130	384 ± 170	0.003
Subfoveal choroidal thickness (μm)	402 ± 115	331 ± 92	3.8 × 10^–5^
Baseline BCVA (log MAR)	0.21 ± 0.37	0.23 ± 0.35	0.62
Descending tract	45 (18.1%)	0 (0%)	8.0 × 10^–4^
Pachydrusen	65 (26.1%)	17 (32.1%)	0.37
Fibrin	16 (6.4%)	3 (5.7%)	0.83
Pigment epithelial detachment	33 (13.3%)	8 (15.1%)	0.72
***CFH* I62V (rs800292)**			
GG	57 (22.9%)	17 (32.1%)	
GA	129 (51.8%)	22 (41.5%)	
AA	63 (25.3%)	14 (26.4%)	
A allele frequency	51.2%	47.2%	0.45
***CFH* rs1329428**			
CC	40 (16.1%)	13 (24.5%)	
CT	138 (55.4%)	24 (45.3%)	
TT	71 (28.5%)	16 (30.2%)	
T allele frequency	56.2%	52.8%	0.52

## Discussion

In the present study, we investigated clinical and genetic differences between sexes in CSC. To the best of our knowledge, this study is the first study to report the different prevalence of descending tracts between sexes in CSC and the age on initial presentation was significantly younger in males compared to females. A previous study analyzing 811 patients reported that males were significantly early-onset compared with females and the peak prevalence for males were 45–49 years and females had two prevalence peak, the higher at 55–59 years and the other 45–49 years^14^. CSC is predominantly seen in middle-aged males compared to females^14^. Previous studies have reported that endogenous testosterone level or exogenous testosterone treatment was associated with CSC^15–17^. These results suggest increased testosterone levels as a possible reason why CSC is frequently seen in males. Therefore, there might be a gender-specific difference of clinical and genetic features in patients with CSC, in addition to the incidence of CSC.

In the present study, the age distribution in males was one peak in the range of 50–54 years and the age distribution in females seems to be two peaks in the range of 50–54 years and over 65 years. Although the age distribution pattern was different from the previous study^14^, the number of the age distribution peaks is the same as in the previous study. The reason why there are two peaks of age distribution in females has not been fully understood. As it has been considered that menopause and the female hormone are the pathogeneses in female patients with CSC, we consider that they are also associated with the onset age of CSC in females.

Descending tracts, a hallmark of the disease chronicity in CSC, were exclusively seen in males in the present study. This finding is characterized on fundus autofluorescence (FAF) as descending areas of hypoautofluorescence with surrounding hyperautofluorescence. Hypoautofluorescence corresponds to the complete RPE and outer retina atrophy (cRORA) moving inferiorly and hyperautofluorescence implicates the presence of previous/current persistent subretinal fluid causing photoreceptor and RPE disruption. The cause of descending tracts is considered to be the RPE vulnerability and RPE pump dysfunction^18^. Although this study is cross-sectional, it might provide us important information. From the present result, RPE vulnerability might be different between sexes in patients with CSC. Recently, Central Serous Chorioretinopathy International Group classified CSC into 2 groups: simple CSC and complex CSC depending on presence or absence of RPE alterations^19^. Although longitudinal studies are needed, male patients might be more likely to progress to complex CSC. However, BCVA was comparable between sexes, it is because a descending tract was foveal-sparing in most eyes.

Compared to female patients, male patients had the greater subfoveal choroidal thickness and lower central retinal thickness. The present study was consistent with previous studies demonstrating a decreased choroidal thickness was seen in female patients or female healthy cohorts^12,13^. Regrading central retinal thickness, we cannot draw a definitive reason why central retinal thickness is greater in females compared with males. We think that chronicity like descending tracts is associated with these results. In the acute phase, subretinal fluid is localized in the macular area in the eyes with CSC. With the progression to the chronic phase, subretinal fluid expanded inferiorly and central retinal thickness decreased accordingly. The prevalence of descending tracts is higher in males compared with females. Therefore, central retinal thickness might be greater in females than in males.

In the genetic point view, the risk allele frequency of *CFH* rs800292 and rs1329428 was not significantly different between sexes. *CFH* is first identified as a variants susceptible to CSC. A Japanese genome wide association study revealed that *CFH* variants were associated with subfoveal choroidal thickness in healthy cohorts. In spite of similar genetic background in *CFH* variants, subfoveal choroidal thickness differed between sexes after adjusting age. This might implicate a gender-difference has a greater effect on subfoveal choroidal thickness^20^.

There are several limitations in this study. The major limitation of the study is retrospective nature of analysis. Second, the proportion of female patients was approximately 17% in the present study. Therefore, female sample size is small although a total number of cohorts was large. Third, axial length was not measured for all eyes. Previous studies demonstrated that axial length is associated with subfoveal choroidal thickness along with age. To confirm and refute the present result, it is necessary to measure axial length for all eyes and apply axial length as a variable. Recently it has been proposed that the sclera thickness is one of the pathogenesis of CSC. The thick sclera might cause the obstruction of vortex vein, resulting in vortex vein congestion leading to choroidal thickening, especially outer choroid^21^. In the present study, we did not measure the sclera thickness. Further studies are necessary to investigate whether the sclera thickness differ according to gender. Although the vertical and horizontal scans through the fovea were performed for all eyes, volume scans covering the whole macula were not performed for all eyes. There was a possibility that we have missed the findings such as shallow irregular PED located on juxta-fovea although the possibility is low.

In summary, female CSC patients were significantly older with a thinner subfoveal choroidal thickness compared with male patients on the initial presentation. Descending tracts might be a gender-specific finding in eyes with CSC.

## Methods

### Ethics comment

This study adhered to the tenets of the Declaration of Helsinki. This retrospective study was approved by both Institutional Review Board/Ethics committees including University of Yamanashi and Kyoto Prefectural University of Medicine. The approved number is 2205. The committees waive the requirement for obtaining informed consent, given that this was a retrospective study of medical records and retrospective registered.

A retrospective medical chart review was performed for consecutive 302 patients diagnosed as central serous chorioretinopathy (CSC) between April 2016 and December 2021 the Macular Clinic, University of Yamanashi and Kyoto Prefectural University of Medicine.

Inclusion criteria consisted of all patients diagnosed as CSC by clinical exam and multimodal imaging and older than 20 years of age. In eyes with CSC, serous retinal detachment is typically seen in the posterior pole on color fundus photography and SD-OCT, FA shows the single/multiple leakage at the level of RPE. ICGA usually shows the choroidal vein dilation and choroidal vascular hyperpermeability on early and late phase, respectively. However, these findings are not inclusion criteria because some proportion in eyes with CSC do not show pachychoroid characteristics. Exclusion criteria included (1) pregnancy as fluorescein and indocyanine green angiography (FA/ICGA) is not typically performed on these patients and (2) presence of shallow irregular pigment epithelial detachment (PED) which could be indicative of type 1 neovascularization as optical coherence tomography angiography was not performed for all participants and (3) patients with a history of topical or systemic steroid use.

**Figure 3:**
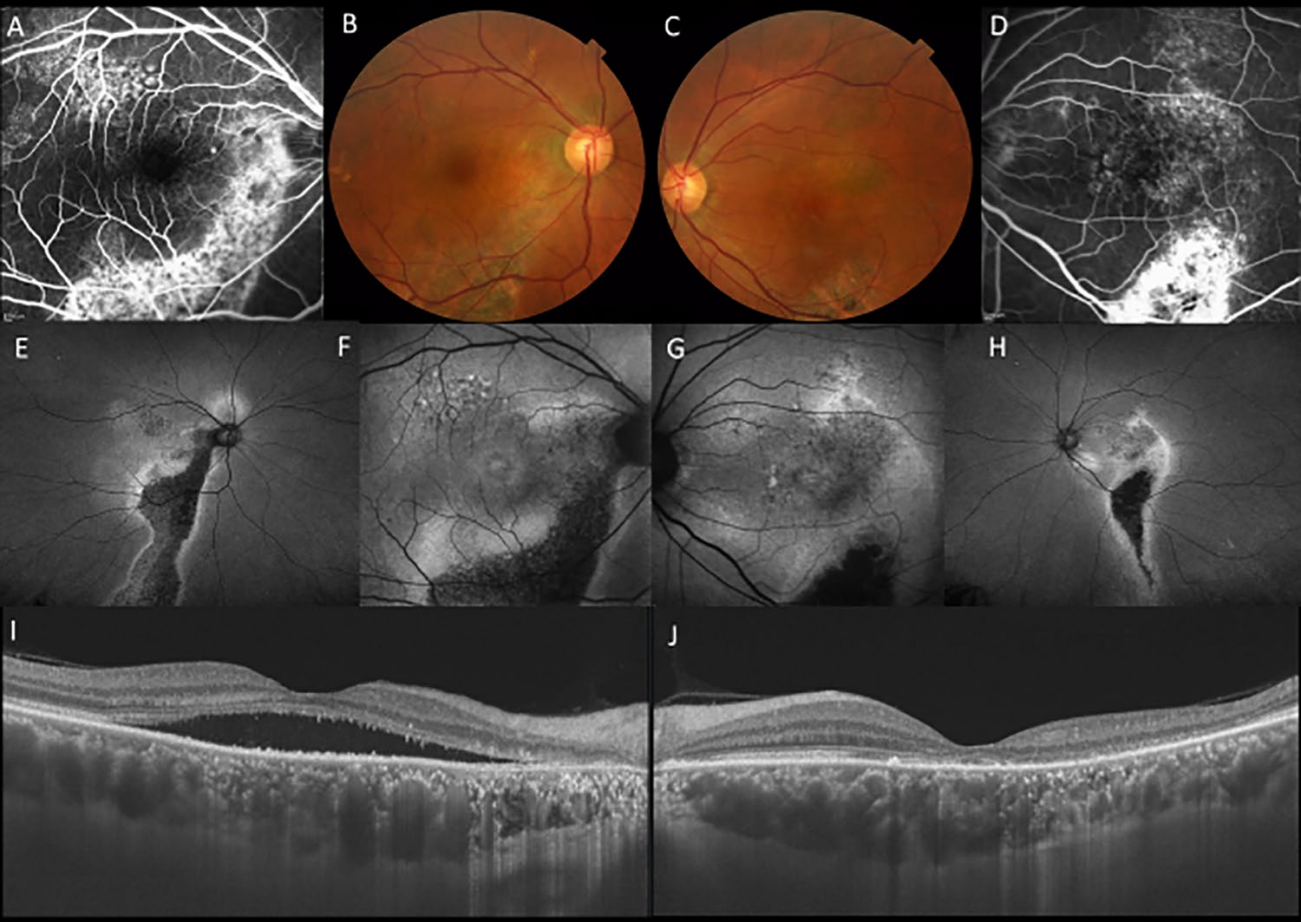
A 56-year-old male patients with central serous chorioretinopathy in both eyes. (**A**) In the right eye, staining was seen along the inferior arcade vessel by fluorescein angiography (FA). (**B**) In the right eye, color fundus photography showed retinal pigment epithelial atrophy in the area corresponding to staining on FA. (**C**) In the left eye, color fundus photography color fundus photography showed retinal pigment epithelial atrophy inferior to the macula. (**D**) In the left eye, staining was seen at the inferior arcade vessel by FA. (**E**) Widefield fundus autofluorescence clearly showed descending tract extending inferiorly in the right eye. (**F**) Fundus autofluorescence suggested the presence of exudation in hyperfluorescence area in the right eye. (**G**) Fundus autofluorescence suggested the presence of exudation or previous exudation in hyperfluorescence in the left eye. (**H**) Widefield fundus autofluorescence clearly showed descending tract extending inferiorly in the left eye. (**I**) Swept-source optical coherence tomography (SS-OCT) showed presence of subretinal fluid and a dilated choroid in the right eye. (**J**) SS-OCT also showed a dilated choroid with resolution of subretinal fluid in the left eye.

On the initial presentation, all patients received comprehensive ophthalmic examination including measurement of best-corrected visual acuity (BCVA) using Landolt chart and intraocular pressure as well as slit-lamp biomicroscopy with or without 78 diopter lens, fundus photography, fundus autofluorescence (FAF) using Optos California/Daytona, FA/ICGA using a confocal scanning laser ophthalmoscope (HRA2, Spectralis, Dossemheim, Germany) and SS-OCT (Atlantis /Triton, Topcon, Tokyo, Japan) or SD-OCT (Spectralis). The diagnosis of CSC was made on the presence of subretinal fluid and leakage from RPE during FA. Eyes without subretinal fluid showing hyperfluorescence on FAF were defined as having a previous history of CSC (Fig. 3).

In eyes with CSC, subfoveal choroidal thickness was measured manually as a vertical distance from Bruch’ membrane to choroidoscleral-border. Central retinal thickness was also measured for all cases and defined as the vertical distance between Bruch membrane and inner limiting membrane. The values in the most recent onset eye were applied if both eyes were affected. If the most recent onset eye was unknown, the values in the right eye were applied in the study. Presence or absence of clinical findings in CSC was independently evaluated based on a multimodal imaging by two retinal specialists (S.Y or Y.S/ A.F or N.T).

**Figure 4:**
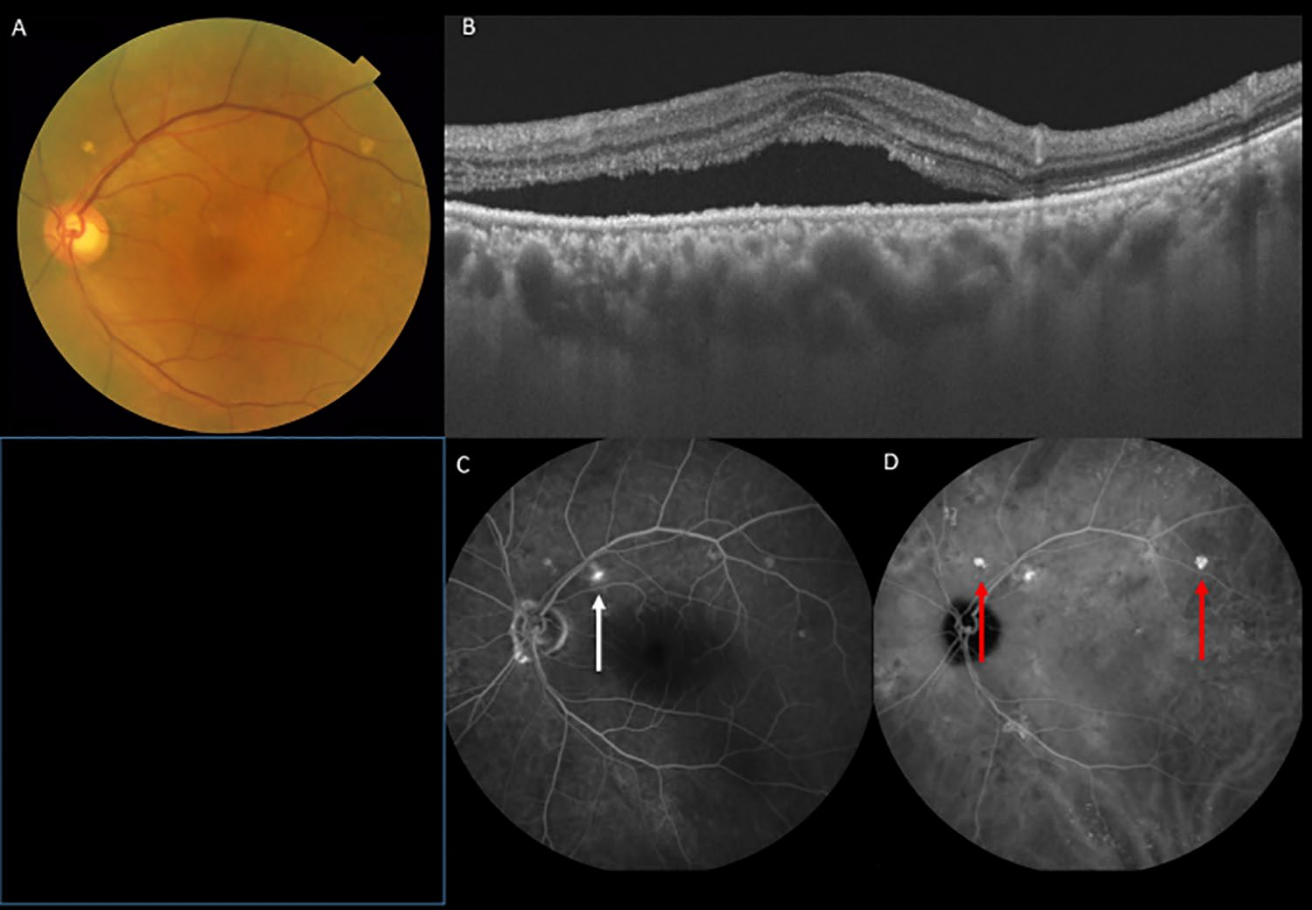
A 70-year-old male patient with central serous chorioretinopathy and pachydrusen in the left eye. (**A**) Fundus photography shows pachydrusen outside the arcade and serous retinal detachment in the macula in the left eye. (**B**) Swept-source optical coherence tomography demonstrated serous retinal detachment and a dilated choroid in the left eye. (**C**) Fluorescein angiography revealed the leakage point indicating by a white arrow in the left eye. (**D**) Late phase indocyanine green angiography revealed hyperfluorescent areas corresponding to pachydrusen indicating by red arrows in the left eye.

**Figure 5:**
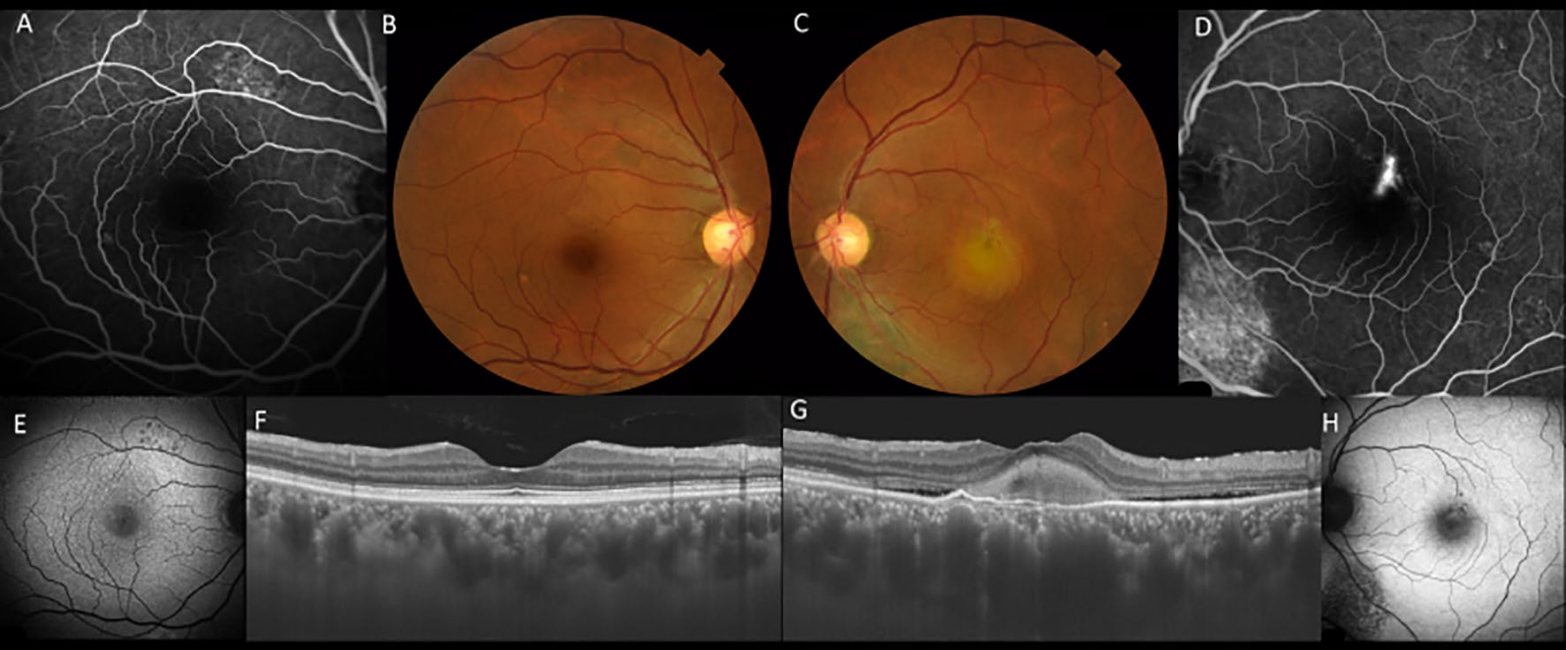
A 48-year-old male patient with central serous chorioretinopathy and fibrin in the left eye. (**A**) Retinal pigment epithelial changes were seen superior to the macula by fluorescein angiography (FA). (**B**) There were no obvious abnormal findings in the right eye. (**C**) A white round lesion was seen along with retinal pigment epithelial atrophy inferior to the optic disc in the left eye. (**D**) A pinpoint leakage and retinal pigment epithelial atrophy inferior to the optic disc was seen by FA in the left eye. (**E**) Fundus autofluorescence (FAF) shows hypofluorescent spots corresponding to retinal pigment epithelial changes in FA in the right eye. (**F**) Swept-source optical coherence tomography (SS-OCT) shows dilated choroid without exudation in the right eye. (**G**) SS-OCT shows hyperreflective materials with shallow serous retinal detachment in the left eye. (**H**) FAF shows hyperautofluorescent corresponding to fibrin area in the left eye.

Pachydrusen were defined as isolated or scattered yellowish drusenoid deposits exceeding 125 µm. Based on late phase ICGA, the lesion corresponding to pachydrusen exhibited hyperfluorescence as previously described (Fig. 4)^22^. Descending tract was defined as descending complete or incomplete outer retinal and/or RPE atrophy corresponding to hypoautofluorescence with surround hyperfluorescence on FAF (Fig. 3). Fibrin was defined as white round lesion on color fundus photography and subretinal hyperreflective material above RPE on OCT (Fig. 5). Dome-shaped pigment epithelial detachment is defined as PED width larger than 500 µm. Discordant diagnosis was resolved through open arbitration if their diagnoses were different.

### Genotyping

A peripheral blood was collected from all participants when FA/ICGA was performed. Genomic DNA was obtained from peripheral blood using PURE GENE ISOLATION KIT (Gentra Systems, Minneapolis, US). The genotyping of rs800292 and rs1329428 was performed for all participants using TaqMan Assays on ABI 7300/7500 Real Time PCR System (Applied Biosystems, Foster City, US) as we previously described^23^. Written informed consent was obtained from each patient regarding genetic analysis.

### Statistical analysis

Statistical analyses were performed using DR. SPSS (IBM, Tokyo, Japan). Differences of continuous variables between 2 groups were evaluated by Mann–Whitney U test and differences of categorical variables between 2 groups were evaluated by chi-square tests. A p-value less than 0.05 were considered a statistical significance.

## Data Availability

All data generated or analyzed during this study are included in this article. Further enquiries can be directed to the corresponding author.
